# Lateral Geniculate Nucleus Volume Assessment Using Linear Mixed Model in Moderate and Advanced Retinitis Pigmentosa

**DOI:** 10.3390/jcm15145665

**Published:** 2026-07-19

**Authors:** Katarzyna Nowomiejska, Anna Niedziałek, Katarzyna Toborek, Aleksandra Czarnek-Chudzik, Robert Rejdak, Radosław Pietura

**Affiliations:** 1 Department of General and Pediatric Ophthalmology, Medical University of Lublin, 20-079 Lublin, Poland; aleksandra.czarnek-chudzik@umlub.edu.pl (A.C.-C.); robert.rejdak@umlub.edu.pl (R.R.); 2 Department of Radiography, Medical University of Lublin, 20-093 Lublin, Poland; anna.niedzialek@umlub.edu.pl (A.N.); radoslaw.pietura@umlub.edu.pl (R.P.); 3 Center for Artificial Intelligence and Computer Modeling, Maria Curie-Skłodowska University, 20-031 Lublin, Poland; katarzynatoborek@gmail.com

**Keywords:** retinitis pigmentosa, lateral geniculate nucleus, 7 Tesla MRI

## Abstract

**Purpose:** We aimed to compare the volume of the lateral geniculate nucleus (LGN) in patients with different stages of retinitis pigmentosa (RP) with regard to age, sex and symmetry of the LGN. **Methods:** The investigated cohort included 13 patients with moderate (median Snellen visual acuity 0.75) and 18 patients with advanced (median Snellen visual acuity 0.06) RP-related visual field loss. The volumes of the left and right LGNs were manually measured using ITK-SNAP software after an examination of the brain with a 7 Tesla MRI. A linear mixed statistical model was used to assess LGN volume regarding age and gender of moderate and advanced RP patients and symmetry of both LGNs. **Results:** The mixed-effects linear model did not reveal a significant effect of disease group on LGN volume after adjusting for age and sex (F(1.27) = 0.01, *p* = 0.91). A significant effect of the LGN side was demonstrated, with the volume of the right LGN being significantly greater than that of the left (F(1.29) = 29.45, *p* < 0.001) in both disease groups, left–right. The interaction between disease group and LGN side was not statistically significant (F(1.29) = 0.45, *p* = 0.51). There is a tendency for LGN to decrease with age (F(1.27) = 3.84, *p* = 0.060), and there is no gender predilection (F(1.27) = 0.11, *p* = 0.74) in RP patients. There was correlation found between left LGN volume and visual acuity (ρ = 0.64) and central retinal thickness (ρ = 0.71) in the moderate group). **Conclusions:** No significant differences in LGN volume were found between patients with moderate and advanced RP. Furthermore, the volume of the right LGN was larger than the volume of the left LGN in RP patients, and this asymmetry is not gender-dependent. Correlation was found between the left LGN volume and visual acuity and central retinal thickness in moderate group. Our findings may have clinical implications for future RP management.

## 1. Introduction

Retinitis pigmentosa (RP) represents a heterogeneous group of inherited disorders characterized by the progressive loss of visual function due to retinal degeneration. These conditions arise from genetically driven dysfunction in retinal morphogenesis, phototransduction, and cellular maintenance pathways, involving photoreceptors, the retinal pigment epithelium, and glial systems [1,2,3]. RP is considered the most common inherited retinal dystrophy globally, which is a rare disease, affecting 1 in 4000 individuals [4].

Night blindness, decreased peripheral vision, and progressive visual field constriction are common clinical manifestations of RP that can manifest at any stage of life, from early childhood to adulthood. In the advanced stage of the disease, patients can become completely blind. This long-lasting disease may be classified into three stages: early, middle, and end stages. In the early stage, night blindness is the main symptom. In the mid-stage, visual field testing shows mild peripheral scotomas that tend to enlarge towards the extreme periphery and macular area. In the end stage, patients can no longer move autonomously, as a result of peripheral vision loss (classical tunnel vision), with few degrees of remaining visual field [4,5,6].

The current treatments for RP include pharmacological approaches, gene therapy, cell transplantation, retinal prostheses, and optogenetics [7,8,9,10]. However, for RP subjects with low visual acuity, conventional visual function examinations, such as visual acuity and visual field assessments, are unreliable and insensitive because of their poor vision and fixation; thus, they may be insufficient for future clinical trials [11,12]. Little is known about the capacity of the adult visual system to process restored visual input after many years of deprivation.

The lateral geniculate nucleus (LGN) is a small, bilateral structure of the thalamus and is considered to be the principal recipient of retinal ganglion cell afferent neurons within the visual pathway. It is located on the dorsolateral aspect of the thalamus, and it can be divided into six clearly visible layers, labeled 1–6 from ventral to dorsal. It regulates the flow of visual information, ensuring that the most important information is sent to the visual cortex [13]. However, it is difficult to identify the LGN on routine 1.5 Tesla magnetic resonance imaging (MRI) images due to low resolution [14]. LGN volume has previously been measured with a 7 Tesla MRI in RP patients, and it was significantly decreased in RPGR-related RP [15], Usher syndrome [16] and advanced RP [17]. However, the specific contributions of the retina and thalamus to LGN properties are presently unknown [18] and the volume of LGN in less advanced stages of RP is also unknown at this time. Because LGN is tiny and has complex layered structures, previous studies primarily evaluated patients with advanced or late-stage RP (where severe volume reduction is already established) [17]. The exact trajectory of how structural atrophy scales during the moderate phase is not fully understood. There is also a lack of widespread correlation data linking specific clinical parameters (such as optical coherence tomography [OCT] parameters and visual field deficits) with structural LGN volumetric changes in moderate RP patients.

The aim of this study was to compare the volume of LGN in two groups of RP patients with moderate and advanced disease stages, using a linear mixed-effects model adjusted for age and sex. The scientific hypothesis is that in patients with moderate RP, the LGN is decreased less than in those with advanced RP.

## 2. Materials and Methods

### 2.1. Patients

This research was approved by the local medical ethics committee of the Medical University of Lublin (KE-0254/246/2020) and was carried out in compliance with national legislation and the Declaration of Helsinki. Patients were recruited in the outpatient clinic of the Department of General and Pediatric Ophthalmology of the Medical University of Lublin, Poland. The 7 Tesla scans of the brain were obtained at the Ecotech Complex in Lublin, Poland. Inclusion criteria were as follows: clinical diagnosis of advanced RP based on characteristic phenotypes and age over 18 years. Clinical features of RP are as follows: night blindness (nyctalopia) and peripheral visual field constriction. Characteristic fundus findings are as follows: bone-spicule pigment deposits, retinal arteriole attenuation, and a waxy pale optic disc, as well as severely diminished or absent rod-driven responses (a-wave and b-wave) in an electrophysiology examination. Overall, 31 patients with a clinical diagnosis of RP were selected at the Department of General and Pediatric Ophthalmology at the Medical University of Lublin, Poland. All RP patients underwent complete ophthalmological examination—best corrected visual acuity (Snellen charts), kinetic visual field examination (Octopus, Haag-Streit, Bern, Switzerland), fundus examination with widefield fundus photography (Optos, Dunfermline, UK) and optical coherence tomography (OCT) examination (Topcon, Tokyo, Japan).

**Figure 1:**
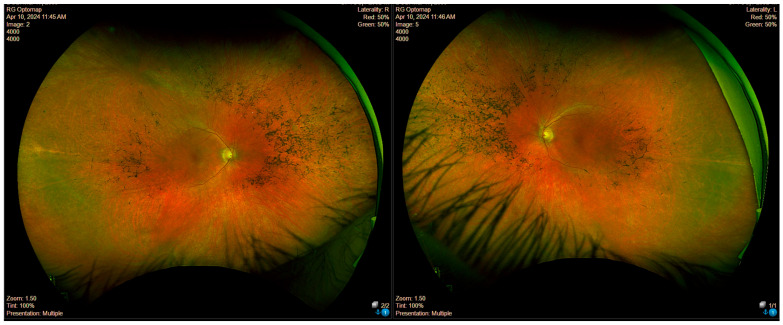
Widefield fundus photography of a patient with moderate retinitis pigmentosa.

**Figure 2:**
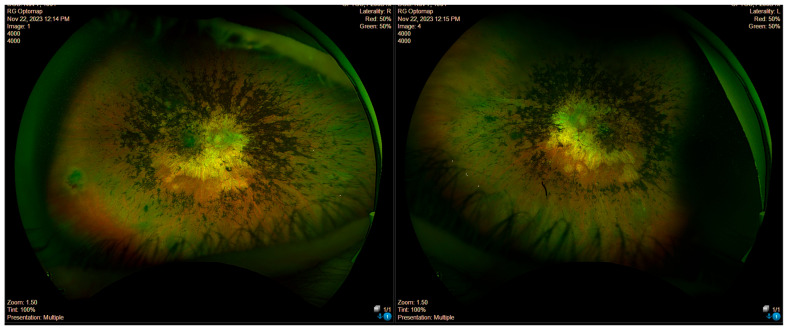
Widefield fundus photography of a patient with advanced retinitis pigmentosa.

This group included 13 patients with moderate RP (Figure 1) and 18 with advanced RP (Figure 2). Patients in the advanced RP group fulfilled criteria of blindness described in the International Classification of Diseases 11th Edition (ICD11), defining blindness as a distance visual acuity worse than 3/60 or a visual field radius less than 10 degrees [19]. Patients in a moderate stage were classified as having three stages of RP based on fundoscopy proposed by Hamel [20]. A moderate (mid) stage was described as bone spicule-shaped pigment deposits present in the mid-periphery along with retinal atrophy, while the macula is preserved, although with a peripheral ring of depigmentation, retinal vessels are attenuated (Figure 1). An advanced stage is described as pigment deposits present all over the retina, very thin retinal vessels, and a pale optic disc (Figure 2).

Baseline demographic and ophthalmological characteristics are presented in Table 1.

Since the assumption of a normal distribution was not met for some of the analyzed variables, the relationships between the volume of the lateral geniculate nucleus (LGN) and ophthalmic parameters were assessed using Spearman’s nonparametric rank correlation coefficient. The strength of the correlation was interpreted according to Evans’ classification (0.20–0.39—weak; 0.40–0.59—moderate; 0.60–0.79—strong).

Patients with advanced disease were significantly older than those in the moderate disease group. The average age in advanced RP was 56.6 years (SD = 12.46), while in the group with moderate RP, it was 29.12 years (SD = 6.44). This difference was statistically significant in Welch’s t-test (t = 7.99, df = 26.72, *p* < 0.0001).

The observed age difference between the groups was significant both statistically and clinically, and there was little overlap between the age ranges. Age was therefore a potential confounding factor in the assessment of the group effect. Additionally, moderate differences in gender distribution between the groups may have influenced the analyzed results. For this reason, both age and gender were included as covariates in all statistical models, regardless of their significance in the final model.

Men predominated in the advanced RP group (61.11%), while women and men were more evenly distributed in the moderate RP group (53.85% women, 46.15% men). Although these differences were not extreme, they justify the inclusion of gender as a control variable in multivariate analyses. Both age and gender were included as covariates in all statistical models, regardless of their significance in the final model.

### 2.2. MRI Examination

MRI examinations were performed at the ECOTECH COMPLEX in Lublin, Poland, using a Discovery MR950 7 T scanner (GE Healthcare, Chicago, IL, USA). The system was equipped with gradients providing a maximum strength of 50 mT/m and a slew rate of 200 T/m/s. Radiofrequency transmission was achieved using a quadrature-driven two-channel birdcage coil, whereas signal reception was performed with a 32-channel phased-array head coil (Nova Head, 2Tx/32Rx, Siemens, Munchen, Germany). The imaging protocol for the present study included a three-dimensional, non-contrast, magnetization transfer-weighted SILENT sequence. Acquisition parameters are summarized in Table 2.

The 3D MT-weighted SILENT sequence provides a magnetization transfer contrast that enhances the visualization of small brain structures, including the lateral geniculate nucleus (LGN), while enabling low-acoustic-noise imaging, effective fat suppression, and a relatively short acquisition time.

### 2.3. Data Analysis

The LGN volume was determined by manual segmentation using ITK-SNAP software (version 4.0.0-rc.2), which enables three-dimensional delineation of anatomical structures in MRI datasets. Automated segmentation was not feasible because of the small size of the LGN, its complex anatomy, and its indistinct boundaries on MRI images. As histopathological confirmation was not available, the true anatomical volume of the LGN could not be determined.

Prior to the formal analysis, the three observers jointly reviewed 7T MRI examinations from 10 subjects to establish a standardized segmentation protocol and improve measurement reproducibility. During this calibration stage, a consensus was reached regarding the identification of the anatomical boundaries of the LGN. Manual segmentation was subsequently performed independently by the three observers using ITK-SNAP in multiplanar reconstruction mode. The LGN was delineated slice-by-slice according to its characteristic anatomical location and signal intensity on the 3D MT-weighted SILENT images, with verification in the axial, coronal, and sagittal planes. Whenever the boundaries were difficult to distinguish, the predefined consensus criteria established during the calibration session were applied.

To minimize observer bias, all MRI datasets were anonymized before image analysis. The observers were blinded to the participants’ clinical diagnoses and all identifying information throughout the segmentation process. Each observer independently segmented the left and right LGN, and the final LGN volume for each subject was calculated as the mean of the three independent measurements. To ensure consistency across observers, all evaluations were performed using identical image display settings (window level: 1000; window width: 1500; minimum intensity: 250; maximum intensity: 1750) on the same GE Advanced Workstation equipped with a calibrated BARCO medical-grade display.

After the completion of all segmentations, the anonymized results were matched to the corresponding participants by an independent researcher who was not involved in the image analysis.

### 2.4. Statistical Analysis

Statistical analyses were performed using linear mixed models. A two-tailed significance level of α = 0.05 was adopted in all statistical tests. Calculations were performed in the R environment (version 4.5.1 (13 June 2025). Linear mixed models were estimated using the lme4 package (version 2.0-6), and the significance of fixed effects was assessed using the lmerTest package (version 3.2-0). Estimated marginal means were calculated using the emmeans package (version 2.0.0).

The following linear mixed model was used:

$${volume}_{ij}=\beta_{0}+\beta_{1}{group}_{i}+\beta_{2}{LGN}_{ij}+\beta_{3}\left({group}_{i}\times {LGN}_{ij}\right)+\beta_{4}{age}_{i}^{c}+\beta_{5}{gender}_{i}+b_{0i}+\varepsilon_{ij}$$

where

*volume_ij_*—volume measured in the j-th LGN in the i-th patient, j ϵ {1,2}, i ∈ {1, 2…, 31};
*β*_0_—free term (average volume in the reference group, for the left eye, at the average age and reference gender);
*group_i_*—disease group (binary variable; reference level: M);
*LGN_ij_*—LGN (left/right; reference level: left);
*group_i_* × *LGN_ij_*—group × eye interaction;
*agei^c^*—age centered on the sample mean;
*gender_i_*—gender (reference level: K);
*b*_0*i*_—random intercept for patient N(0, σ_id^2);
*ε_ij_*—random component at observation level N(0, σ^2).

The analysis covered 62 observations from 31 RP patients, which corresponds to two measurements per person (the volume of the left and right lateral geniculate nuclei (LGN) was measured in each patient). Due to the nested data structure resulting from two measurements (left and right LGN) in each patient, a linear mixed model was used for the analysis. The model included a random intercept for each patient, which allowed for the correlation between measurements taken from the same person to be taken into account (i.e., the model does not treat measurements from the left and right LGN of the same patient as independent observations but takes into account the fact that they are similar to each other because they come from the same person). The dependent variable was the volume of the LGN measured in mm^3^. Fixed effects included disease stage (moderate vs. advanced RP), LGN side (left vs. right), the interaction between disease stage and LGN side, age, and sex. Model parameters were estimated using restricted maximum likelihood (REML). Associations between the mean LGN volume and mean best-corrected visual acuity (BCVA) were evaluated separately in the moderate and advanced RP groups using Spearman’s rank correlation coefficient.

**Figure 3:**
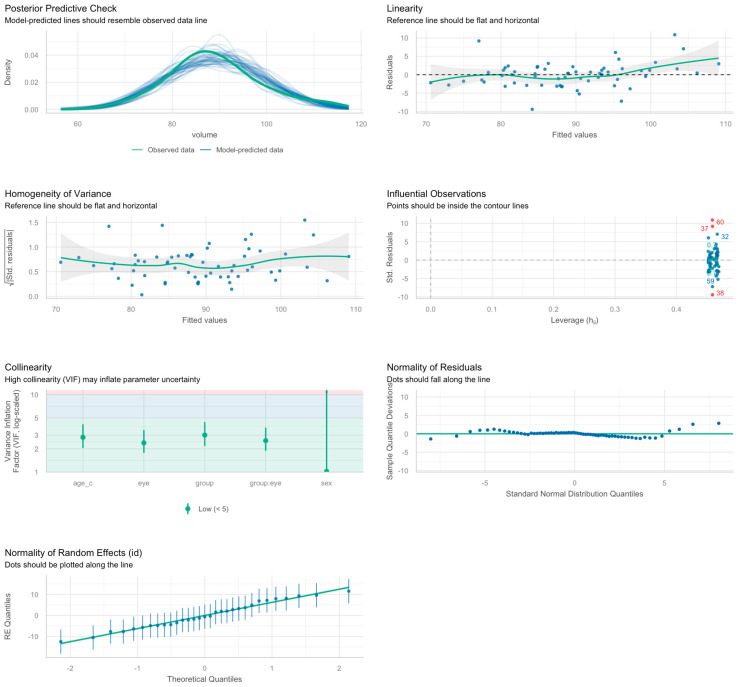
Diagnostic plots for the linear mixed-effects model, including posterior predictive checks, residual-versus-fitted plots, homogeneity of variance, influence diagnostics, collinearity assessment, normal Q–Q plots of residuals, and Q–Q plots of random effects.

Model assumptions were assessed by visual inspection of residual and normal Q–Q plots. No substantial violations of model assumptions were observed. Diagnostic plots are included in Figure 3.

## 3. Results

A linear mixed-effects model was used to evaluate the effects of disease stage, LGN side, age, and sex on LGN volume. The results of the fixed-effects analysis are presented in Table 3.

**Figure 4:**
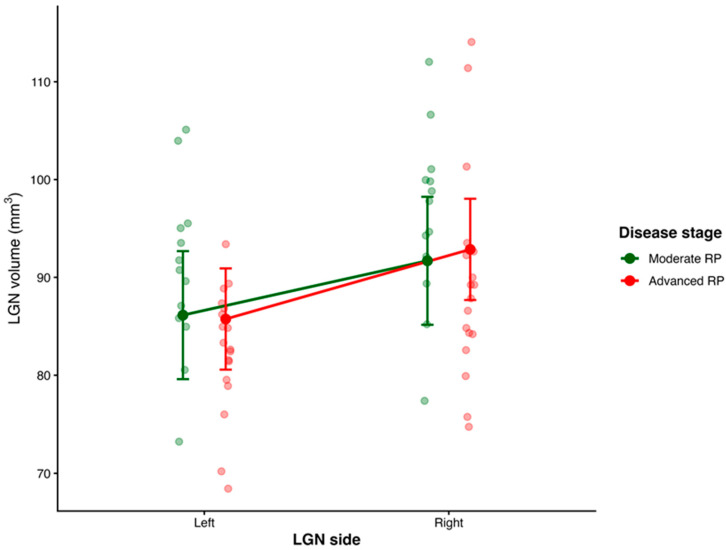
Estimated average marginal lateral geniculate nucleus (LGN) volumes (95% CI) by side and disease group, obtained from a linear mixed model adjusted for age and gender.

Patients with moderate and advanced RP showed comparable adjusted LGN volumes. In both groups, the estimated volume of the right LGN was consistently greater than that of the left LGN (Figure 4).

For the left LGN, the mean volume was 86.1 (SE = 3.27) in the moderate group and 85.9 (SE = 2.61) in the severe group. For the right LGN, these values were 91.6 (SE = 3.27) and 93.0 (SE = 2.61), respectively.

No significant effect of disease group on volume was found (F(1.27) = 0.01, *p* = 0.91). However, a significant effect of side was demonstrated—the volume of the right LGN was significantly greater than that of the left (F(1.29) = 29.45, *p* < 0.001).

Age was associated with a decline in LGN volume (F(1.27) = 3.84, *p* = 0.060), while gender had no significant effect on the variable under study (F(1.27) = 0.11, *p* = 0.74).

Estimated age- and sex-adjusted marginal means indicate similar volume values between disease groups for both the left and right LGN. Higher volume values were observed in the right LGN in both groups (Table 4).

Positive correlations were found between left LGN volume and BCVA in the left eye (ρ = 0.64; *p* = 0.018) for moderate RP and the OCT parameter (central retinal thickness—CRT) of the left eye (ρ = 0.71; *p* = 0.007). As the sample size for the visual field analyses (n = 23) was lower because visual field data were unavailable for eight participants, we did not include it in the analysis (Table 5).

## 4. Discussion

In the present study, analysis using a linear mixed model, considering the nested data structure (left and right LGN measurements in the same patient), showed no significant differences in LGN volume between patients with moderate and advanced RP. This result persisted after adjustment for age and gender, indicating that the observed lack of group effect is not due to demographic confounding. At the same time, the existence of a clear and statistically significant left–right LGN asymmetry was confirmed, with the right LGN being larger than the left. This effect was stable and independent of disease severity, confirming the absence of a significant interaction between disease group and the LGN side. This means that the observed asymmetry is general in nature and does not change with disease progression. It has already been reported that the volume of the left LGN is smaller than the right one in patients suffering from glaucoma [21,22]. Kosior-Jarecka and colleagues [21] as well as Lee and co-authors [22] used manual delineation of LGN, the same as in our study. In different stages of glaucoma, LGN volume values from early glaucoma differed significantly from the advanced group, but not from controls [22].

LGN was first detected in vivo using MRI with proton density (PD)-weighted images by Horton et al. in 1990 [23]. LGN is a part of the visual pathway, spanning from the retina to the primary visual cortex, which comprises anatomically and functionally distinct regions that serve as potential loci for pathological disruptions leading to visual impairments. Anatomically, the LGN receives retinal nerve fiber from ipsilateral temporal retinal ganglion cells (RGCs) and contralateral nasal RGCs. Due to the partial crossing of nerve fibers in the visual pathway (optic chiasm), there is no direct “one eye—one LGN” relationship. This approach reflects the overall performance of the visual system and avoids the need to perform multiple parallel correlation analyses, which could increase the risk of a type one error. Our results indicate a correlation between left LGN volume and CRT in OCT within both RP groups analyzed. In patients with glaucoma, no significant correlation was found between visual function (visual field sensitivity) and LGN volume [24].

In RP degeneration of rod photoreceptors, the cells controlling night vision precede and exceed cone degeneration, as a majority of RP genetic mutations affect rods selectively. There is evidence that the retina progressively reduces its ability to transmit visual information to the brain via the optic nerve. Damage to retinal ganglion cells in the retina leads to degeneration of the visual cortex (anterograde degeneration) [24]. The underlying mechanisms of this process, known as trans-synaptic degeneration (TSD), are unknown. It has already been proven that there is a reduction in white matter volume in the brain in RP patients [25]. Since the neurodegenerative process is already biologically advanced by the time symptoms appear, having biomarkers available in the preclinical phase to signal that the pathological process is in progress is essential to obtain effective therapies, even more so if the biomarkers are sensitive to novel therapeutic treatments, such as gene therapies or retinal implants [26]. These interventions rely on the inner retina and visual cortex retaining enough healthy neural architecture to process the visual signals.

The patients’ age showed a weak, marginally significant correlation with LGN volume, suggesting a tendency for it to decrease with age. Although this effect did not reach conventional statistical significance, its direction is consistent with the biologically expected impact of aging on central nervous system structures and may be significant in a larger sample size. It has already been proven by 1.5 Tesla MRI that the human LGN shows an approximately 15% reduction in structural volume between 20 and 70 years of age [27].

Gender was not a significant predictor of LGN volume after accounting for other variables in the model, suggesting that the observed differences in volume are not gender-dependent in the study population.

We can anticipate that neuroimaging methods might be useful in the future to evaluate the degree of recovery in blind patients who accept novel methods of treatment, such as gene therapy or retinal implants. However, restoring appropriate function to the retina using these methods does not necessarily imply that the patients can see again, given that plasticity of the primary visual cortex retained by the adult brain is limited, especially after many years of blindness. It has already been shown that participants with RPE65 mutations showed intact visual pathways using fMRI, which became responsive and strengthened after gene therapy treatment [28,29,30,31]. Moreover, after surgery, implanting the Argus II implant, six out of seven RP blind subjects were able to detect high-contrast stimuli using the prosthetic implant. Before the implant, the Blood Oxygenation Level-Dependent (BOLD) activity in V1 and the lateral geniculate nucleus (LGN) was very weak or absent. Surprisingly, after prolonged use of Argus II, BOLD responses to visual input were enhanced [32]. The exact underlying mechanism of these changes has not been identified in experimental studies. In a study using 3 Tesla MRI in 10 patients after gene therapy due to RPE65 mutations, it was shown that retinal gene therapy promotes robust expansion and an increase in LGN volume [33]. The authors conclude that LGN is the key thalamic nucleus responsible for direct communication between the retina and the visual cortex and it undergoes structural plasticity in response to retinal gene therapy. Moreover, the observed LGN volume increase was positively correlated with the clinical measures of the patients’ visual fields, particularly evident from the correlations of the left LGN with the left and right eye visual fields, similar to our study. Furthermore, in a study of LGN in advanced RP, left LGN volume was correlated with visual acuity [17]. The pattern of reduced gray matter volume in visual primary and association cortices was significantly correlated with the extent of the peripheral visual field deficit in a cohort of 27 RP patients [29]. The weakness of our study is that not all RP patients were genotyped. However, RP is an enormously heterogeneous disease, with more than 100 genes involved [34] and this fact may also affect the LGN volume results. We already investigated RPGR-related RP patients [15] and other cohorts of RP patients are planned to be examined with 7Tesla MRI. Other potential shortcomings are: slight non-linearity for large predicted values, mild deviations from normality in the tails of the residual distribution, and individual observations that may be influential. These risks appear to be moderate, but they should be taken into account when interpreting the results or conducting a sensitivity analysis. The diagnostics are visual in nature; subtle violations of assumptions may not be fully detected. The conclusions relate to the quality of the fit, rather than causality or stability of the estimates beyond the range of the observed data.

We noted relatively large variability in mean BCVA within the advanced RP group, suggesting the presence of heterogeneous visual functions. As inclusion criteria for the advanced group were visual acuity less than 0.1 or visual field constricted to less than 10 degrees of radius, there was quite a large range of values of visual acuity. Another shortcoming of the study is that the sample size (n = 31) is quite small; however, RP is a rare disease with a prevalence of less than 1 in 2000 of the population. The number is similar to previous studies with RP or glaucoma patients examined with 7 Tesla MRI [13,15,16,17].

To the best of our knowledge, this is the first study using 7 Tesla MRI to compare different groups of visual impairment within a cohort of RP patients. As already proven in publications [15,16,17], LGN volume is decreased in comparison to an age-matched control group in patients with advanced RP, RPGR-related RP and Usher syndrome. We aimed in the present study to compare LGN volume in moderate and advanced groups of RP patients. The hypothesis that LGN volume decreases less in less advanced stages of RP is not supported by our findings. However, the absence of significant differences between moderate and advanced RP does not necessarily prove preserved plasticity or a stable visual pathway structure. It may reflect a limited sample size, age imbalance, disease heterogeneity, measurement variability, or insufficient staging granularity. We also found asymmetry between left and right LGN volume, with the right LGN appearing larger than the left one. Our findings offer a deeper insight into the LGN structure in RP, revealing the potential neural mechanisms underlying visual impairment in retinal dystrophies. The findings establish that RP extends beyond the eye, actively causing measurable anterograde trans-synaptic atrophy within the visual pathway. By utilizing 7 Tesla MRI, researchers can overcome the historically low resolution of standard MRI to visualize the LGN and observe how retinal damage shrinks it. Moreover, we identified a correlation between left LGN volume and visual function and structure in RP patients. Further studies are required to explore the relationship between LGN volume and visual function, as well as the volume of other brain structures in RP patients.

## Figures and Tables

**Table 1:** Descriptive statistics of age, visual acuity, visual field and OCT parameters according to disease severity.

Variable	Group	Mean ± SD	Median	Min	Max	N
Age (years)	Advanced RP	56.6 ± 12.46	57.5	35	74	18
	Moderate RP	29.2 ± 6.44	30.0	20	42	13
BCVA left eye	Advanced RP	0.11 ± 0.12	0.07	0.01	0.40	18
	Moderate RP	0.76 ± 0.13	0.80	0.60	1.00	13
BCVA right eye	Advanced RP	0.09 ± 0.09	0.07	0.01	0.30	18
	Moderate RP	0.71 ± 0.16	0.70	0.30	0.90	13
Mean BCVA	Advanced RP	0.10 ± 0.10	0.06	0.01	0.30	18
	Moderate RP	0.73 ± 0.12	0.75	0.55	0.95	13
Radius of visual field OP (°)	Advanced RP	10.00 ± 0.00	10.0	10	10	10
	Moderate RP	36.92 ± 11.09	30.0	20	60	13
Radius of visual field OL (°)	Advanced RP	10.00 ± 0.00	10.0	10	10	10
	Moderate RP	37.69 ± 10.13	40.0	20	50	13
CRT in OCT OP (µm)	Advanced RP	225.44 ± 34.93	226.0	159	299	18
	Moderate RP	244.85 ± 43.54	240.0	163	358	13
CRT in OCT OL (µm)	Advanced RP	226.89 ± 23.83	226.0	188	284	18
	Moderate RP	250.77 ± 27.88	250.0	195	303	13

Data are presented as mean ± standard deviation (SD), median, minimum, maximum, and sample size (N). Visual field analyses were based on all available observations. Due to unavailable data caused by nystagmus or poor visual acuity, visual field measurements were available for 10 patients with advanced RP and 13 patients with moderate RP, whereas OCT and BCVA measurements were available for 18 and 13 patients, respectively. BCVA = best-corrected visual acuity; OP = right eye; OL = left eye; OCT = optical coherence tomography; CRT = central retinal thickness.

**Table 2:** Acquisition parameters of the 3D MT-weighted SILENT sequence.

Parameter	3D MT-Weighted SILENT
Acquisition time	6 min 30 s
Field of view (FOV)	17.6 × 17.6 cm
Slice thickness	0.8 mm
Echo time (TE)	0.0 ms
Repetition time (TR)	257 ms
Matrix size	224 × 224
Number of excitations (NEX)	3
Flip angle (FA)	2°

**Table 3:** Fixed-effect estimates from the linear mixed-effects model, presented as regression coefficients (β) with 95% confidence intervals.

Fixed Effect	Estimate (β, mm^3^)	95% CI	*p*-Value
Disease group (Advanced vs. Moderate)	−0.23	−10.23 to 9.77	0.963
LGN side (Right vs. Left)	5.56	1.99 to 9.13	0.003
Group × Side	1.57	−3.12 to 6.25	0.506
Age (Per Year)	−0.28	−0.56 to 0.01	0.055
Sex (Male vs. Female)	−0.96	−6.74 to 4.82	0.740

**Table 4:** Estimated marginal means (EMMs) obtained from a linear mixed model for the right and left lateral geniculate nucleus (LGN) in patients with medium and advanced retinitis pigmentosa (RP), adjusted for age (centered variable) and gender. Means, standard errors (SEs) and 95% confidence intervals are given.

Group	LGN	EMM	SE	95% CI—Upper	95% CI—Lower
Moderate stage RP	left	86.1	3.27	92.7	79.4
Advanced stage RP	left	85.9	2.61	91.2	80.5
Moderate stage RP	right	91.6	3.27	98.3	85.0
Advanced stage RP	right	93.0	2.61	98.3	87.7

**Table 5:** Spearman’s rank correlation coefficients between lateral geniculate nucleus (LGN) volume and ophthalmic parameters stratified by group (BCVA—best corrected visual acuity; RP—retinitis pigmentosa; CRT—central retinal thickness; OCT—optical coherence tomography).

Variable	Group	LGN	Spearman’s ρ	*p*
BCVA right eye	Advanced RP	Right	0.26	0.299
	Moderate RP	Right	−0.08	0.803
CRT in OCT right eye	Advanced RP	Right	0.48	0.049
	Moderate RP	Right	0.34	0.255
BCVA left eye	Advanced RP	Left	0.15	0.578
	Moderate RP	Left	0.64	0.018
CRT in OCT left eye	Advanced RP	Left	0.36	0.144
	Moderate RP	Left	0.71	0.007

## Data Availability

The original data presented in the study are openly available at https://doi.org/10.18150/NMP4BG, accessed on 24 April 2026.
